# Nucleolin interacts with influenza A nucleoprotein and contributes to viral ribonucleoprotein complexes nuclear trafficking and efficient influenza viral replication

**DOI:** 10.1038/srep29006

**Published:** 2016-07-04

**Authors:** Olivier Terrier, Coralie Carron, Benoît De Chassey, Julia Dubois, Aurélien Traversier, Thomas Julien, Gaëlle Cartet, Anaïs Proust, Sabine Hacot, Denis Ressnikoff, Vincent Lotteau, Bruno Lina, Jean-Jacques Diaz, Vincent Moules, Manuel Rosa-Calatrava

**Affiliations:** 1 Virologie et Pathologie Humaine - Team VirPath - Université Claude Bernard Lyon 1 - Hospices Civils de Lyon, Lyon, France; 2 CIRI, International Center for Infectiology Research, Inserm U1111, CNRS UMR5308, ENS Lyon, Université Claude Bernard Lyon 1, Lyon, France; 3 VirNext, Faculté de Médecine RTH Laennec, Université Lyon 1, Lyon, France; 4 Centre de Recherche en Cancérologie de Lyon, UMR Inserm 1052 CNRS 5286, Centre Léon Bérard, Lyon, France and Université de Lyon, Lyon, France; 5 CIQLE, Centre d’imagerie quantitative Lyon-Est, Université Claude Bernard Lyon 1, Lyon, France; 6 Hospices Civils de Lyon, Laboratory of Virology, Lyon, France

## Abstract

Influenza viruses replicate their single-stranded RNA genomes in the nucleus of infected cells and these replicated genomes (vRNPs) are then exported from the nucleus to the cytoplasm and plasma membrane before budding. To achieve this export, influenza viruses hijack the host cell export machinery. However, the complete mechanisms underlying this hijacking remain not fully understood. We have previously shown that influenza viruses induce a marked alteration of the nucleus during the time-course of infection and notably in the nucleolar compartment. In this study, we discovered that a major nucleolar component, called nucleolin, is required for an efficient export of vRNPs and viral replication. We have notably shown that nucleolin interacts with the viral nucleoprotein (NP) that mainly constitutes vRNPs. Our results suggest that this interaction could allow vRNPs to “catch” the host cell export machinery, a necessary step for viral replication.

Influenza A viruses (IAV) are responsible for respiratory diseases that affect millions of people worldwide in recurrent annual epidemics, sometimes fatally. The ability of this major human pathogen to mutate and lead to the emergence of new pandemic subtypes, constitutes a constant serious threat to human health^1^. The genome of IAV consists of eight single-stranded negative RNA segments encoding for up to 17 distinct proteins^2^. These RNA segments are encapsidated by the viral nucleoprotein (NP) in association with proteins of the viral polymerase complex (PA, PB1 and PB2). The resulting viral ribonucleoproteins (vRNPs) constitute autonomous functional units for viral transcription and replication^1^,^3^.

In contrast with most RNA viruses, IAV have a nuclear infectious cycle and thus have direct access to the host nuclear machineries, including the transcriptosome or the spliceosome, to successfully achieve their viral replication^2^,^3^,^4^. Numerous cellular components have been identified as functional interacting partners of IAV proteins and some are subject to different extents of viral “retasking”^3^,^5^,^6^,^7^,^8^,^9^. In view of their nuclear replication, IAV require the efficient export of their vRNPs from the nucleus towards the cytoplasm and budding regions of the cell membrane for a productive infection^10^. Extensive investigation of the nuclear export of vRNPs has provided a model in which the viral matrix protein (M1) binds to vRNPs and the viral nuclear export protein (NEP/NS2) acts as a bridge between M1 and the cellular export receptor Crm1^11^. Alternatively, NP has been shown to bind to both M1 and Crm1 thereby allowing its exportation^12^. Nevertheless, deciphering the precise functional interplay between IAV and the host Crm1-dependent nuclear export machinery requires further exploration. Recently, Chase and collaborators showed that the interaction between vRNPs and the nuclear export machinery takes place in dense chromatin domains that allow IAV to “snatch” Crm1-RanGTP nuclear export complexes to the detriment of cellular substrates^13^. These results, together with other observations, highlight the involvement of host nuclear compartmentalization and chromatin territories in regulating nuclear traffic of IAV vRNPs^14^,^15^,^16^,^17^.

Several investigations, including ours, have revealed that IAV induce a strong remodeling of nuclear architecture with marked modifications of the nucleoli ultrastructure and compartments^3^,^17^,^18^,^19^,^20^. The nucleolus, known as the site of ribosome biogenesis, is also a sensor of cellular stresses and is involved in several cellular pathways, such as cell-cycle regulation or apoptosis^21^,^22^,^23^. Several DNA and RNA viruses, including cytoplasmic viruses are known to induce nucleolar alterations that contribute towards optimal infection^24^,^25^,^26^.

In the context of IAV, their interplay with nucleolus and selective interactions with several nucleolar components appears to be decisive in the outcome of infection^17^,^20^. Ozawa and colleagues demonstrated that dynamic nucleolar localization of NP is functionally significant by contributing to efficient viral replication and transcription^27^. Moreover, different studies have demonstrated a subtype dependent interaction between viral non-structural protein (NS1) and cellular nucleolin, suggesting a role of the nucleolar targeting functions of NS1 in IAV pathogenesis^28^,^29^. Proteomics-based approaches have revealed changes in the nucleolar proteome of IAV infected cells^5^,^30^ and identified several nucleolar components, such as nucleolin, nucleophosmin (B23) and ribosomal proteins, as putative interactants of reconstituted IAV vRNPs^7^. In addition, high-throughput RNAi approaches have highlighted the functional requirement of several of these nucleolar components in viral replication^31^,^32^,^33^.

Nucleolin is a multifunctional protein that contributes extensively to DNA and RNA regulatory mechanisms, including ribosome biogenesis, chromatin remodeling, mRNA stability and translation, nuclear export of RNA and protein complexes, and microRNA processing^34^,^35^. In this way, nucleolin takes part in several cellular functions, such as gene silencing, senescence, cell proliferation and growth^23^,^35^,^36^,^37^, and continuously shuttles between nucleolar/nuclear compartments and the cytoplasm up to the cell surface^38^,^39^. Moreover, through its ability to associate with diverse target RNAs, nucleolin is implicated in various pathological processes, particularly cancer and viral infection^40^.

In this context, different functional interactions with nucleolin have been described for several DNA viruses, such as poliovirus^41^, adenovirus^42^, HSV^43^,^44^ or CMV^45^,^46^, and several RNA viruses, such as hepatitis C and delta viruses^47^,^48^, coronavirus^49^, hPIV-3 50, papillomavirus HPV9 51, Crimean–Congo hemorrhagic fever virus^52^, HIV1 53, noroviruses^54^ and notably the infectious salmon anemia virus (ISAV) which belongs to the family *Orthomyxoviridae,* as IAV^55^. Altogether, these studies highlight nucleolin as a key interacting factor that is crucial, in some cases, for viral replication and involved in nucleo-cytoplasmic export of viral genome or viral egress^44^.

At first, we observed the dynamic redistribution of nucleolin from a nucleolar to a very specific nuclear polarized pattern during infection. The aim of this study was therefore to investigate the functional relationship between IAV and nucleolin with regards to the nuclear traffic of vRNPs and their targeting to the dense chromatin compartment^13^ and in the context of the viral remodeling of the host nuclear architectures^17^,^20^. We first characterized NP, the main component of IAV vRNP, as co-localizing and interacting with endogenous nucleolin during infection. Moreover, using cell fractionation and RNAi approaches, we suggest that nucleolin contributes to efficient vRNP trafficking and viral replication. Based on our results, we propose nucleolin as an essential host factor for the chromatin targeting of the nucleocytoplasmic export complex of vRNPs during infection.

## Results

### Nucleolin redistributes with IAV ribonucleoprotein complexes to the nuclear periphery of infected cells

**Figure 1:**
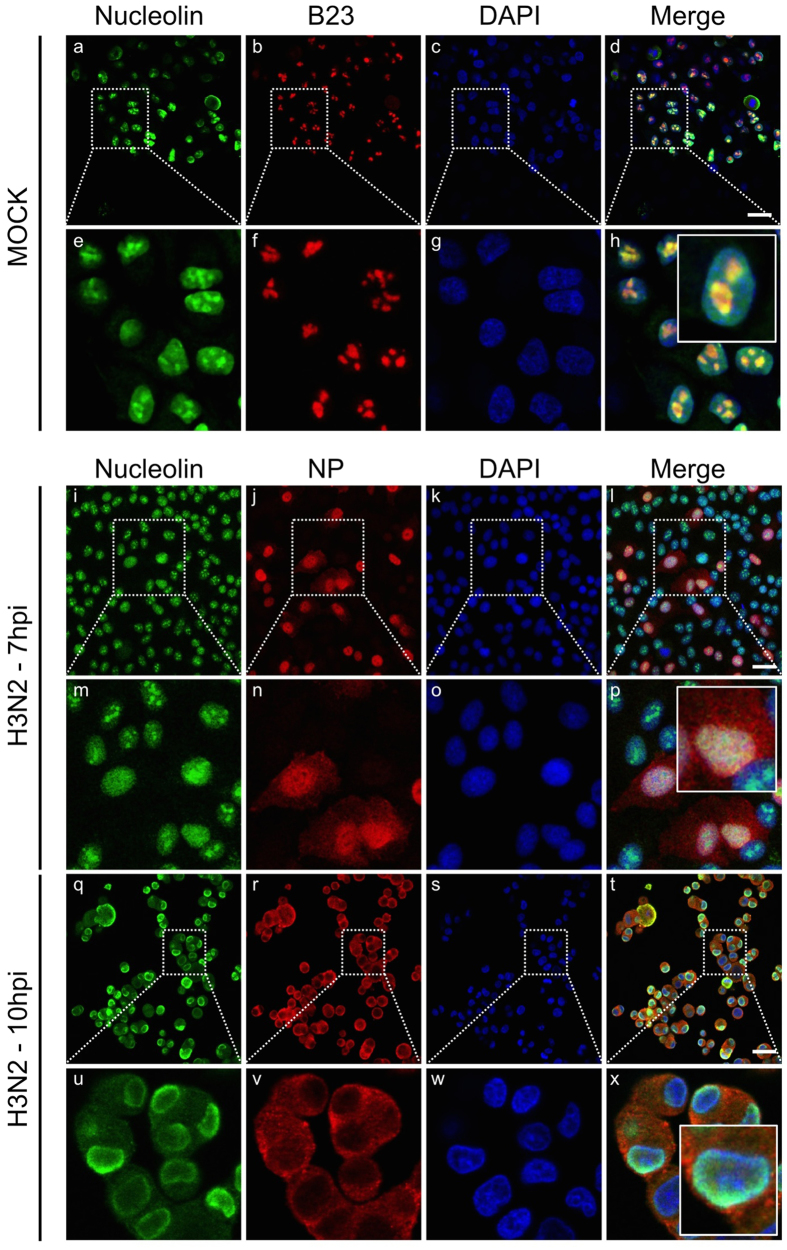
Host nucleolin is dynamically relocalized during the course of influenza infection. Immunofluorescence staining of nucleolin (green) and B23 (red) in mock-infected cells (panels a to h) or NP (red) in A549 cells infected with H3N2 virus at a MOI of 1 (panels i to x) was performed at different times, as indicated. Nuclei were counterstained with DAPI (blue, panels c,g,k,o,s,w). Merged fluorescent signals are presented in panels d,h,i,p,t and x. Cell details are enlarged (inset). Scale bar = 10 μm.

Considering the strongly altered host nucleolar ultrastructure and composition during IAV infection, we analyzed the subcellular localization of three major constitutive nucleolar proteins, nucleolin, B23 and fibrillarin, during the time course of influenza infection. We used immuno-fluorescence confocal microscopy in human lung epithelial A549 cells, mock-infected or infected by influenza A/Moscow/10/99 (H3N2) virus, at 7 and 10 hours post infection (hpi). In addition to nucleolin, B23 or fibrillarin immunostaining, we also used NP as an indicator of viral cycle advancement, as previously carried out^3^,^17^,^18^,^19^,^20^. As expected in mock-infected cells, the three nucleolar proteins mainly localized in nucleoli (Fig. 1, panels a to h and Supplementary Figure 1B,C). During infection, localization patterns of B23 and fibrillarin remained unmodified up to 7 hpi (data not shown) compared to mock-infected cells, as previously reported^7^,^27^. In contrast, when NS1 is notably accumulated within nucleolus at this time (Supplementary Figure 1A), nucleolin no longer preferentially localized in the subnuclear compartment and instead displayed a broad relocalization throughout the nucleus (Fig. 1, panels i to p; and Supplementary Figure 1A), in accordance with previous study^28^. Later in infection (10 hpi), as illustrated by the mainly cytoplasmic localization of NP, nucleolin mostly redistributed at the nuclear periphery of more than 95% of the infected cells (Fig. 1, panels q to x). At this time most of the fibrillarin accumulated in a large nuclear spot (Supplementary Figure 1B), probably linked to the ultrastructural remodeling of the nucleolar compartment^3^,^17^,^18^,^19^,^20^, whereas B23 remained associated (Supplementary Figure 1C).

Altogether, these results reveal the differential modification of the subnuclear localization of the three abundant constitutive nucleolar proteins by H3N2 infection, and suggest a specific, earlier and dynamic redistribution of nucleolin during the time course of infection.

We then investigated the impact of infection on nucleolin localization by a different human (H1N1 pdm2009) and several avian (H5N1, H5N2 and H7N7) IAV strains. Despite their specific host-range restriction^56^, all viruses efficiently infected A549 cells with comparable kinetics, as we previously reported^3^,^17^. We performed immunolabeling at 24 hpi, a time corresponding to late stage of infection for all these viruses (Supplementary Figure 2A). As observed for H3N2, each infection led to a similar redistribution of endogenous nucleolin towards the nuclear periphery of infected cells (Supplementary Figure 2A). Interestingly, the observed subnuclear localization pattern of nucleolin in IAV infected cells clearly evoked the previously described polarized distribution of trafficking vRNPs that formed a cap at the apex of the nuclear envelope prior to their export towards the cytoplasm^57^,^58^. To note, similar NP-nucleolin colocalization and subnuclear localization patterns were also observed using another specific monoclonal antibody raised against NP (MAb 3/1, kind gift of Dr Webster) that was reported to recognize IAV vRNP complexes^59^ (Supplementary Figure 2B).

**Figure 2:**
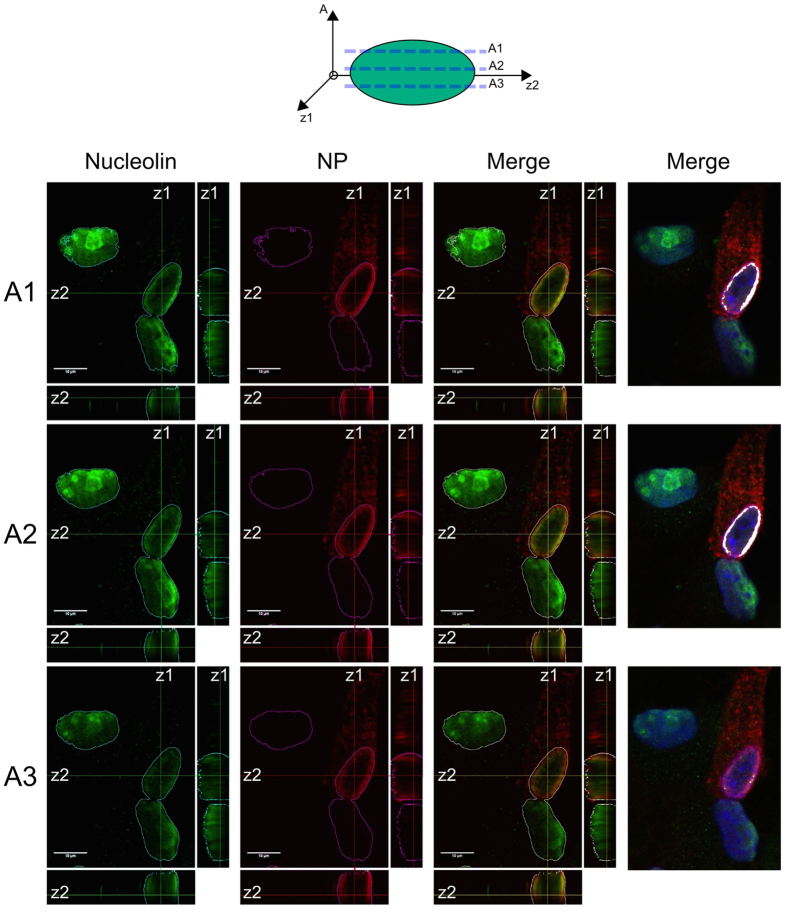
Influenza virus induces a nuclear polarized redistribution of nucleolin. A549 cells were infected with H3N2 (MOI 1) and fixed at 8 hpi. Immunofluorescence staining of NP (red) and nucleolin (green) were performed. Single optical sections through the top (**A1**), the midline (**A2**) and the bottom (**A3**) of nuclei (xy) and z-axis reconstruction (z1 and z2, respectively) are represented. Merged fluorescent signals are presented, as indicated. Scale bar = 10 μM. JAcoP plugin for imageJ was used to highlight the colocalization of the two stainings by thresholding the signal above the background^80^. For easier visualization, colocalized pixels are colorized in white (right panels).

To further investigate these correlated observations, we studied the localization of endogenous nucleolin by 3D reconstruction of confocal images. Our observations confirmed an asymmetric localization pattern of endogenous nucleolin within H3N2 infected cells (10 hpi) (Fig. 2). Single optical sections through the nucleus (Fig. 2, panels A1 to A3) and Z-axis reconstruction revealed a polarized co-localization of nucleolin with NP towards the nuclear apex, as revealed by the asymmetric merged fluorescence staining in the z1-z2-axis plane (Fig. 2). Altogether, these results suggest a putative involvement of nucleolin in the nuclear trafficking of vRNPs during IAV infection.

### Endogenous nucleolin inter**a**cts with transiently expressed NP

**Figure 3:**
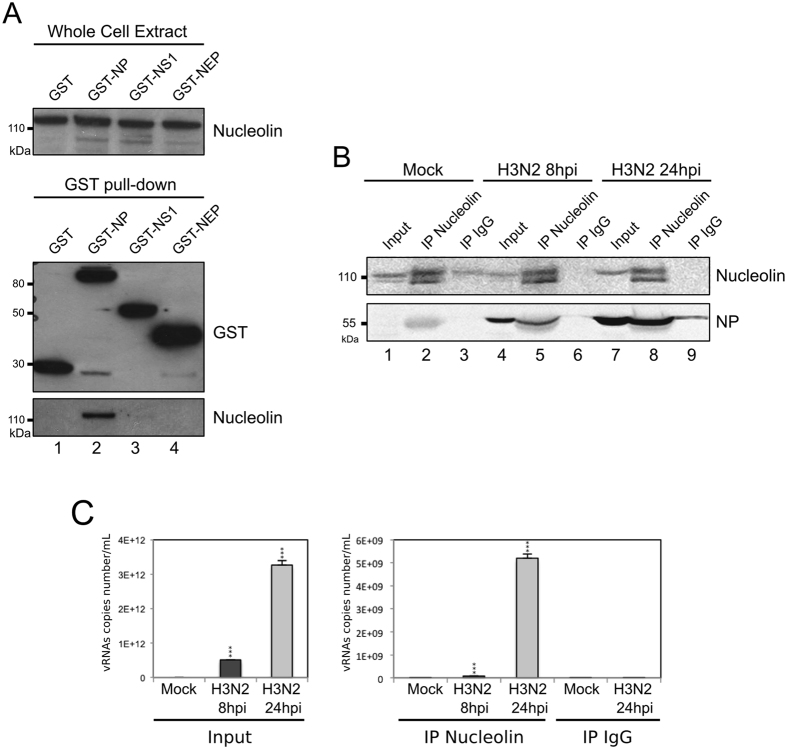
NP interacts with endogenous nucleolin (**A**) A549 cells were transfected with plasmids coding GST or, alternatively, GST fused to NP, NS1 or NEP. Cell lysates were subject to a pull-down assay. Whole cell extracts and pull-down proteins were resolved on SDS-PAGE and immunoblotted with anti-GST and anti-nucleolin antibodies. Top-panel shows detection of the endogenous nucleolin within whole cell lysates. The bottom panel shows the level of expression of pulled-down GST fusion and endogenous nucleolin. **(B)** Cells were infected during 8 or 24 hours with H3N2 (MOI 2). Immunoprecipitations (IP) were performed on the whole cell extract with polyclonal anti-nucleolin or control IgG antibodies, as indicated. The presence of nucleolin and NP in immunopurified complexes was checked by western blot analysis. Whole cell extracts (Input) were used as control. **(C)** Cells were infected during 8 or 24 hours with H3N2, as before. Immunoprecipitations were performed with the corresponding whole cell extract proteins, using polyclonal anti-nucleolin or control IgG antibody. RNAs associated with purified complexes were extracted and M vRNAs were quantified by specific RT-qPCR (M vRNAs copies number/mL). Total RNAs were extracted from Mock- or H3N2-infected cells and used as controls (Input). ***P-value <0.001.

To further explore this hypothesis, we first performed a GST pull-down assay to examine whether endogenous nucleolin could interact with nucleoprotein, the main component of vRNPs. A549 cells were transfected with a plasmid encoding NP fused to GST (GST-NP, H3N2), and cell lysates were pulled down using glutathione sepharose beads at 48 h post transfection. Complexes were analyzed by western blot with anti-GST and anti-nucleolin antibodies. As controls, cell lysates obtained after transfection of non-fused GST, GST-NS1 or GST-NEP were also examined as controls. As shown in Fig. 3A, endogenous nucleolin was pulled down from GST-NP lysates (Fig. 3A, lane 2). In comparison, no interaction with nucleolin was characterized with cell lysate obtained after transfection of non-fused GST protein (Fig. 3A, lane 1). In addition, a very weak interaction with nucleolin was characterized with GST-NS1 (Fig. 3A, lane 3). This partial discrepancy with a previous study^29^ could be explained by the different strain origin of the NS1 tested (A/WSN/33), especially considering other reports showing strikingly different properties among NS1 of various origins^28^. Another explanation would be our experimental conditions being based on transient expression in A549 cells and not on the use of recombinant proteins expressed from bacteria^29^. On the other hand, our use of GST-NEP protein expression as a negative control did confirm no interaction with nucleolin (Fig. 3A, lane 4). Additionally, this interaction between endogenous nucleolin and transiently expressed NP was also confirmed by immunoprecipitation assay using a specific rabbit polyclonal anti-nucleolin antibody^43^ (data not shown).

### Endogenous nucleolin is a putative interacting partner of IAV ribonucleoproteins in infected cells

To determine whether NP association with nucleolin also occurs in a context of infection, we performed immunoprecipitation assays with extracts prepared from A549 cells infected with H3N2 virus, at 8 h and 24 hpi. Extracts from mock-infected cells were used as negative controls. As expected, western blot confirmed the immunoprecipitation of nucleolin specifically by the rabbit polyclonal anti-nucleolin antibody compared to IgG control (Fig. 3B, lanes 2, 5 and 8 *versus* 3, 6 and 9). The immunoprecipitated complexes from infected cells contained NP, the amount of which increased from 8 hpi to 24 hpi (Fig. 3B, lane 5 *versus* 8). Otherwise, RNAse A treatment did not abolished immunoprecipitation of NP, suggesting nucleolin could interact with both vRNP and non-vRNP forms of NP (Supplementary Figure 3). Alternatively, we investigated the presence of vRNA in immunoprecipitated complexes in similar experimental conditions (Fig. 3C). To this end, we subjected cell lysates of infected cells (8 and 24 hpi) to anti-nucleolin immunoprecipitation before extracting the putative associated vRNAs for quantification by specific RT-qPCR (Fig. 3C), described in our previous study^60^. As expected, total cell lysates at 24 hpi contained more vRNA copies as compared to those at 8 hpi; total cell lysates from mock-infected cells contained no detectable vRNA. Accordingly, we detected vRNAs in complex containing NP at 8 hpi and mostly at 24 hpi, whereas no vRNA in control (IgG) (Fig. 3C, right panel).

Altogether, these results demonstrate that NP and vRNA are co-immunoprecipitated with endogenous nucleolin in IAV infected cells. In accordance with previous results of immunostaining experiments and NP interaction assays, our results suggest a putative association of endogenous nucleolin with IAV ribonucleoproteins.

### Silencing of endogenous nucleolin expression impairs nucleocytoplasmic trafficking of IAV ribonucleoprotein complexes

**Figure 4:**
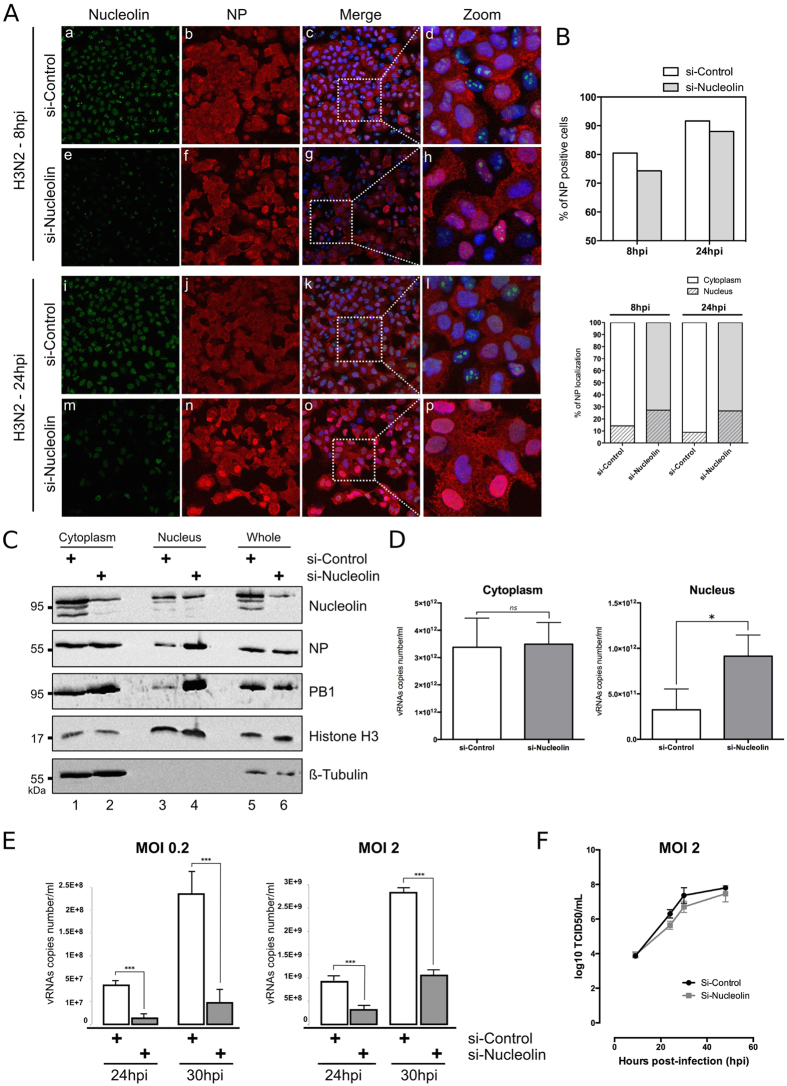
Silencing of endogenous nucleolin alters the nucleocytoplasmic export of vRNPs and decreases the virus production yield. (**A**) A549 cells were transfected with either nucleolin (si-Nucleolin, panels e–h and panels m–p) or control (si-Control, panels a–d and panels i–l) siRNAs. Two days after transfection, cells were infected with H3N2 for 8 h or 24 h and immunostained with anti-nucleolin (green) and anti-NP (red) antibodies. Nuclei were counterstained with DAPI. Panels (c,g,k,o) are merged confocal images. Details of cell are enlarged (inset d,h,l,p). **(B)** Percentage of infection was quantified in si-transfected cells (upper panel). Intranuclear localization of NP was evaluated by counting cells harboring nuclear and/or cytoplasmic labeling in a total of 100 cells per condition, on a total of three distinct fields (lower panel). **(C**,**D)** A549 cells were transfected with si-Nucleolin or si-Control and infected with H3N2 virus, as in A and submitted to nucleocytoplasmic fractionation. Equal amounts of proteins from each fraction were analyzed by western blot (**C**). NP and PB1 proteins were detected using specific antibodies. H3 and β-tubulin were detected as loading and fractionation controls. **(D)** Total RNAs were extracted from cytoplasmic and nuclear fractions and vRNAs were quantified by specific RT-qPCR (M vRNAs copies number/mL), *P-value <0.05. **(E)** A549 cells were transfected with si-Nucleolin or si-Control. Two days after transfection, cells were infected with H3N2 at MOI 0.2 and 2 for 24 to 30 hpi. Viral RNAs were extracted from culture supernatants and quantified by RTqPCR, using specific primers for M segment. ***P-value <0.001. (**F**) A549 cells treated by si-Control or si-Nucleolin (as previously described) were infected with viruses at a MOI of 2. Samples of supernatants were harvested at several time points post-infection and stored at −80 °C until end point viral titration assays in MDCK cells using the Reed and Muench statistical method (TCID50/mL).

We investigated functional relevance of such putative association by studying the contribution of nucleolin to the nuclear trafficking of vRNPs. We first assessed, as an indicator of infection progress, the subcellular distribution pattern of NP in conditions of endogenous nucleolin silencing. After validating silencing efficiency in non-infected A549 cells (Supplementary Figure 4), we transfected A549 cells with siRNA control (si-Control) or a pool of siRNAs targeting nucleolin (si-Nucleolin) before their subsequent infection at 48 h with H3N2 virus (MOI of 1). As expected, the si-Nucleolin treated cells displayed very low levels of nucleolin staining at 8 and 24 hpi, as compared to si-Control (Fig. 4A, panels e and m). Interestingly, the silencing of nucleolin expression appeared to only slightly decrease the number of NP positive cells (Fig. 4A, panels b *versus* f, j *versus* n and Fig. 4B, upper panel), suggesting a limited impact on cell infectivity. In contrast, the si-nucleolin treatment strongly affected the subcellular localization of NP during infection (Fig. 4A, panels b *versus* f, j *versus* n) in such a way that 1.9 to 3 times more cells harbored a stringent nuclear pattern only for NP in this condition, compared to the si-Control condition at 8 and 24 hpi, respectively. At both time intervals post-infection, the viral cycle appeared more advanced in the presence of endogenous nucleolin as shown by a more pronounced cytoplasmic staining for NP in infected cells (Fig. 4B, lower panel).

In a parallel experiment, we assessed by western blot the levels of two protein components of vRNP (NP and PB1) in subcellular compartments (Fig. 4C). A549 cells, previously transfected with si-Control or si-Nucleolin, were infected with H3N2 virus (MOI 1) for 24 h and harvested for nucleocytoplasmic fractionation. As a control, we studied the expression of histone H3 and β-tubulin. As expected, endogenous nucleolin had been silenced in the different sub-cellular fractions (Fig. 4C, compare lanes 1, 3 and 5 with lanes 2, 4 and 6, respectively). Moreover, the two experimental si-RNA conditions showed no difference in terms of expression levels of NP and PB1 viral proteins in whole cell extracts, suggesting no effect on viral protein expression (Fig. 4C, lanes 5 and 6). In contrast, we noted a strong increase of NP and PB1 in the nuclear fractions of si-Nucleolin-treated cells as compared to those of si-Control (Fig. 4C, lanes 3 and 4), while the cytoplasm fractions displayed similar protein ratios between the two si-RNA conditions (Fig. 4C, lanes 1 and 2). We completed this analysis by performing total RNA extraction from the cytoplasmic and nuclear fractions, followed by a quantification of viral genome copy numbers by RT-qPCR (Fig. 4D). Results revealed no difference in cytoplasmic fractions between si-Nucleolin and si-Control (Fig. 4D, left panel). In contrast, the nuclear fraction of si-Nucleolin treated cells contained a significative higher level of vRNA copies than that of the control (Fig. 4D, right panel, p-value <0.05). In accordance with these results, the silencing of nucleolin also significantly reduced the yield of progeny virus produced at 24 and 30 hpi, as compared to si-Control conditions. Quantification of vRNA copies number/ml in cell supernatants revealed a marked decrease in viral progeny yield that could reach up to 95.5% at 30 hpi, (MOI 0.2, Fig. 4E) or up to 63% at 30 hpi (MOI 2, Fig. 4E). This result was also confirmed by quantification of infectious titers (TCID50/mL) in supernatants, with a slight decrease and a delay of viral production in the context of silencing of nucleolin (Fig. 4F).

Altogether, these results suggest a nuclear retention of vRNP complexes upon silencing of endogenous nucleolin and a consecutive delay in the dynamic trafficking of vRNPs towards the cytoplasm and consecutive viral production. They therefore provide evidence in support of our hypothesis that nucleolin is required for an efficient nucleocytoplasmic export of vRNPs during IAV infection.

### Association of vRNPs with dense chromatin domain requires host nucleolin expression

Several reports have demonstrated that vRNPs are associated with chromatin during the nuclear steps of the viral cycle^16^,^61^,^62^,^63^. Recently, Chase and collaborators demonstrated that IAV gain privileged access to the host nuclear Crm1 export machinery by targeting vRNP complexes to the sites of Ran regeneration within dense chromatin^13^. Taking into account our above results together with the functional role of histone chaperone played by nucleolin via its association with chromatin^64^, we examined the contribution of nucleolin towards the observed close association between vRNPs and the dense chromatin compartment.

**Figure 5:**
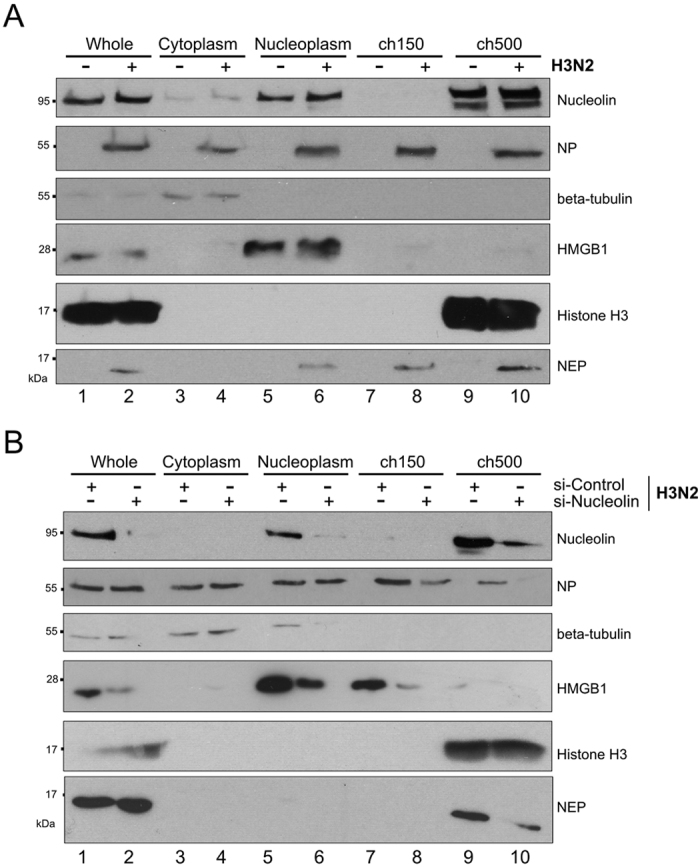
Nucleolin is required for the association of vRNPs with the host chromatin during infection (**A**) A549 cells were infected with H3N2 virus for 24 h (MOI 1) or mock-infected. Fractionated lysates were analyzed by western blot for detection of nucleolin, NP, NEP, and subcellular markers β -tubulin, HMGB1 and histone H3. **(B)** A549 cells were transfected with either si-Nucleolin or si-Control. Two days after transfection, cells were infected with H3N2 for 24 hours (MOI 1). Cells were subfractionated and lysates were analyzed by western blot. H3 and β-tubulin were detected as loading and fractionation control. wh.: Whole fraction; cytoplasm: cytoplasmic fraction; nucleoplasm: nucleoplasmic fraction; ch150: low salt chromatin fraction; ch500: high salt chromatin fraction.

For this purpose, we performed subnuclear fractionation of A549 cells either mock-infected or infected with H3N2 (Fig. 5A). From the initial whole fraction (Whole, lanes 1–2), we harvested cytoplasmic (Cytoplasm, lanes 3–4), soluble nucleoplasmic (Nucleoplasm, lanes 5–6), and the functionally distinct low-salt chromatin (ch150, lanes 7–8) and high-salt chromatin (ch500, lanes 9–10) fractions, according to the protocol described by Chase and collaborators^13^,^65^. We used western blot detection of NP as a marker of vRNP and different subcellular markers, such as β-tubulin (cytoplasm), histone H3 (chromatin), to confirm the successfulness of fractionation (Fig. 5). In accordance with Chase *et al*.^13^,^65^, NP was detected in all cellular fractions, including the ch150 and ch500 fractions at 24 hpi (Fig. 5A, lanes 2, 4, 6, 8 and 10). Interestingly, nucleolin was shared between nucleoplasmic and ch500 fractions in mock-infected cells, whereas at 24 hpi, it had slightly accumulated in the ch500 fraction (Fig. 5A, compare lane 10 *versus* 9) Thus, these results suggest that nucleolin relocalizes to regions of high chromatin density where vRNP export complexes accumulate during infection.

To further support these results, we assessed the association of vRNP components with chromatin under conditions of endogenous nucleolin depletion during infection (Fig. 5B). Again, we infected A549 cells, previously transfected for 48 hours with si-Control or si-Nucleolin, with H3N2 virus. At 24 hpi we performed subnuclear fractionations to detect NP, nucleolin and subcellular markers by western blot, as described above. In the si-Control condition, NP localized in the cytoplasmic, nucleoplasmic and chromatin fractions (Fig. 5B, lanes 1, 3, 5, 7 and 9) and control subcellular markers were detected as expected, whereas nucleolin mainly accumulated in ch500 fraction (Fig. 5B, lane 9). In contrast, effective silencing of nucleolin led to a decrease in the level of NP found in Ch150 fraction a more pronounced decreased in Ch500 fraction at 24 hpi (Fig. 5B, compare lane 8 *versus* 7 and lane 10 *versus* 9), in agreement with the hypothesis of an alteration in the vRNP targeting to chromatin.

Altogether, these results indicate that nucleolin strongly accumulates in regions of high chromatin density during IAV infection and is required for an efficient association of vRNPs with this chromatin compartment during their nuclear trafficking.

## Discussion

Influenza A are nuclear replicating viruses which have direct access to and thus hijack host nuclear machineries in order to achieve optimal infection^3^. In this context, several studies including ours have described the extensive modifications of nuclear ultrastructure and composition^7^,^5^,^30^ with marked nucleolar and chromatin alterations in IAV infected cells^17^,^18^,^20^. In accordance with these observations, we report in this study the differential delocalization through the nucleus of the three major nucleolar components, nucleolin, B23 and fibrillarin during the time-course of IAV infection. Interestingly, the earlier and specific redistribution of nucleolin was of interest and in particular its dynamic colocalization with NP at the vicinity of the inner nuclear membrane, where vRNPs accumulate before their nuclear export^13^,^16^,^58^. Consequently, we then investigated the putative connection between nucleolin and vRNP traffic, notably in regard to a previous study that identified nucleolin as a possible interaction partner of reconstituted IAV vRNPs, without further exploring the functional significance of this interaction^7^. Interestingly, some functional interactions have been described in other viral models, such as feline calicivirus (FCV) and Norwalk virus (NV), in which infections also result in a relocalization of nucleolin from nucleoli to nucleoplasm and the perinuclear area. In this context, nucleolin was shown to act as a positive regulator of viral replication by forming RNP complexes with viral RNA and interacting with the viral NS6/7 proteins^54^.

In this way, our results obtained in GST-pull down and immunoprecipitation assays indicate that endogenous nucleolin interacts with NP and suggest a possible association with vRNP complexes in the context of IAV infection. This hypothesis is supported by several results obtained in conditions of infection such as (i) the relocalization of endogenous Nucleolin in subnuclear compartments in which vRNPs components NP (Fig. 2) and PB1 (Supplementary Figure 2C) are also accumulated, (ii) the detection of vRNA within immunoprecipitated Nucleolin-NP complex (Fig. 3C), and (iii) the silencing of endogenous nucleolin expression that led to an altered nuclear trafficking of vRNPs and a consecutive significant delay in viral production (Fig. 4).

An interesting way to explore should be the role of RNA in the interaction between two RNA binding proteins such as NP and nucleolin. Possibly, the flexibility of NP structure in promoting RNA binding which likely involves conformational change(s) of NP, as previously suggested^66^, would consecutively impact on its interactions with cellular factors.

Altogether, our results play in favor of a possible model in which nucleolin is involved in the nucleocytoplasmic export of vRNPs during IAV infection, even if we cannot exclude concomitant impact on other steps of viral replication (e.g. viral transcription, viral mRNA translation) due to the multifunctional properties of the host nucleolin.

Data from several other studies converge on a model for vRNP traffic illustrated by a vRNP nuclear export complex consisting of as a daisy chain of proteins and involving dynamic and intricate interactions between vRNP, viral and host factors^62^,^67^,^68^,^69^. Previous *in vitro* experiments demonstrated that both vRNPs and M1 interact with the chromatin components at two different sites, RNP to the histone tails and M1 to the globular domain of a histone octamer^62^,^70^, whereas NEP acts as an adapter and interacts with the host Crm1 export machinery^68^.

**Figure 6:**
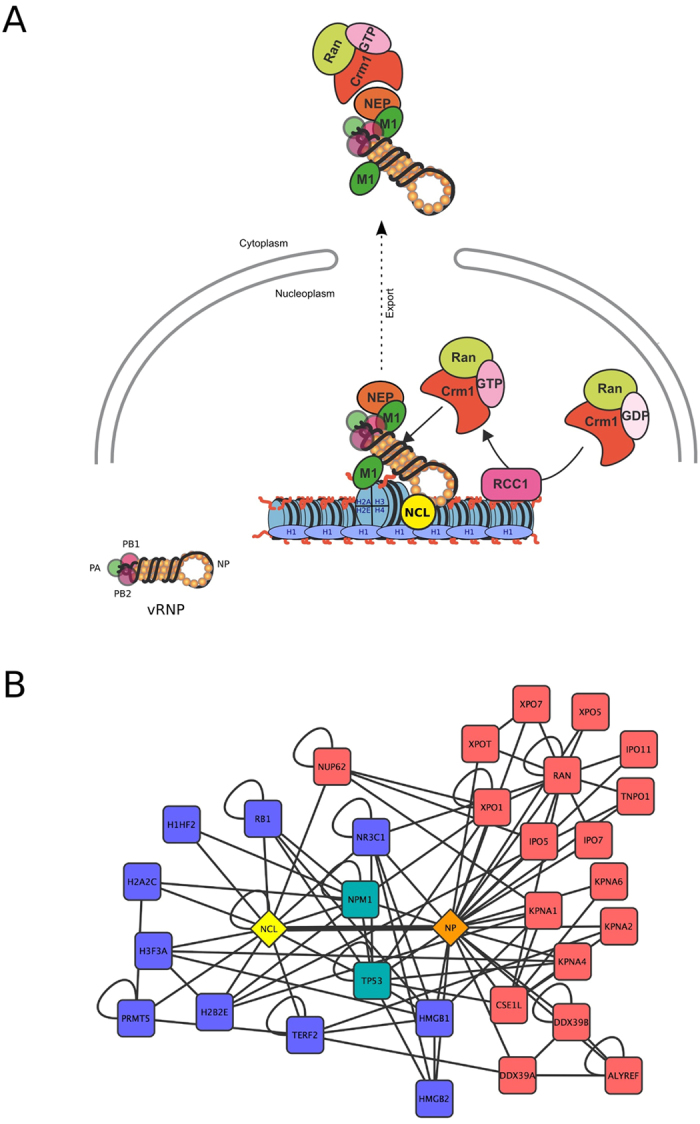
Model for the nucleolin contribution in the vRNPs export at the crossroads between chromatin organization and nucleocytoplasmic transport. (**A**) Influenza viruses gain preferential access to host cell export machinery by targeting chromatin at the sites of Ran regeneration, as proposed by Chase *et al*.^13^. As a chromatin remodeler and an interaction partner of histone H1, nucleolin should contribute to the chromatin targeting of vRNPs. (**B**) The interaction between nucleolin (NCL, yellow diamond) and NP (orange diamond) is placed on the center of the graph (thick edge). Proteins interacting with nucleolin or NP and involved in chromatin organization are in blue squares. Proteins interacting with nucleolin or NP and involved in nucleo-cytoplasmic transport are in red squares. NPM1 (B23) and TP53 involved in both processes are in green squares.

However, several questions remain unanswered, notably what really happens in the nucleus during IAV infection, what are the exact modifications of nuclear architecture and notably how does chromatin remodeling contribute towards vRNP trafficking? Chase and collaborators proposed that association between vRNPs and export machinery occurs in dense chromatin domains, where RCC1 exchanges GDP for GTP to produce export energy. They suggested that IAV gain preferential access to host nuclear export machinery by targeting vRNP export complexes to chromatin-dense territories which confer a physical proximity to the sites of Ran regeneration^13^. Our results support the model proposed by Chase and collaborators and also suggest the requirement of nucleolin in the close association between vRNPs and the host chromatin (Fig. 6A). Indeed, by subnuclear fractionation, we observed a strong accumulation of nucleolin within the condensed chromatin domain (ch500 fraction), which is targeted by vRNPs at 24 hpi. Moreover, depletion of endogenous nucleolin induced a decrease in accumulation of NP, a component of vRNP trafficking through the condensed chromatin fractions.

Our hypothesis relies on the chromatin remodeling and histone chaperone activities of the nucleolin^34^,^64^,^71^. Indeed, nucleolin is known to induce chromatin de-condensation by binding to histone H1^72^ and thus has a function analogous to the family of high-mobility-group (HMG) proteins. In this context, the nucleolin-H1 interaction could cooperate to reorganize cellular chromatin in order to facilitate its efficient targeting by vRNPs. By interacting with NP, the main component of vRNPs, nucleolin would thus constitute a key mediator in the vRNP nuclear export (Fig. 6A). Alternatively and in regards to RNase sensitivity assay, nucleolin most probably also interacts with non-vRNP form of NP during infection, which could incidentally have an impact on several other steps of viral cycle, as well, including vRNP assembly and/or trafficking.

Analysis of enriched gene ontology terms corresponding to the interactants of NP and nucleolin provided molecular support to the pivotal role of NP-nucleolin association in linking chromatin organization and nucleo-cytoplasmic transport biological processes (Fig. 6B). Indeed, a significant proportion of nucleolin interactants are involved in chromatin organization, such as components of nucleosome (*e.g.*: H2A2C, H3F3A; Blue Squares, Benjamini P-value = 4.3 × 10^−2^) or chromatin associated factors such as HMGB1 or HMGB2. Alternatively, NP interactants are strongly enriched in nucleo-cytoplasmic transport proteins, such as RAN or KPNA1, and protein complexes associated with the nuclear envelope such as Nup62 (Red squares, Benjamini P-value = 1.7 × 10^−2^) (Fig. 6B). Interestingly, at the interface of chromatin organization and nucleo-cytoplasmic transport processes, and in addition to p53 (TP53) which we have previously shown to be involved in multiple levels of IAV- host interactions^73^,^74^, B23 (NPM1) represents another common interactant of NP and nucleolin as well as interacting with vRNP^7^,^28^.

Further studies are required to elucidate the mechanism through which nucleolin contributes to IAV vRNP trafficking and to identify other host factors involved in chromatin remodeling and post-translational modifications of histones in the context of IAV infection. Another important consideration is the constant shuttling of nucleolin between subcellular compartments which may reflect a dependence of nucleolin on post-translational modifications that determine its localization^36^. Therefore, the possibility that the phosphorylation and methylation of nucleolin, which influence its interaction with nucleic acids or ADP-ribosylation^40^, also contribute to its nuclear redistribution during IAV infection, remains to be elucidated/investigated.

Beyond the role of nucleolin in IAV vRNP trafficking, its binding to NP could also contribute to viral transcription/replication in accordance with previous observations of nucleolin’s impact on IAV polymerase activity^31^,^32^,^33^. This further hypothesis is consistent with the facts that (i) IAV transcription is dependent on the cellular transcription machinery^61^ and (ii) vRNP binds nucleosomes^62^ and chromatin-associated factors^7^. Similarly to HMGB1 75, putative nucleolin binding to NP/vRNA complexes could facilitate the recruitment of vRNPs at transcriptionally active sites of chromatin and also contribute in this way to efficient IAV replication.

Additionally, the NP-nucleolin association could also restrict nucleolar functions of nucleolin (ribosome biogenesis, ribosome transport) and thereby contribute to the strong remodeling of nucleolar compartment observed during IAV infection^17^,^18^,^20^. Concomitantly, by directly binding to nucleolin in the nucleolus^28^,^29^, NS1 may contribute towards nucleolar disruption resulting in the subsequent redistribution and availability of nucleolin for vRNP trafficking and viral replication. However, further studies are needed to investigate the specific sequence of events occurring in such a viral hijacking of nucleolin. An interesting approach could be to further investigate the histone mimic of NS1 which was recently reported to be able to recruit several host chromatin-associated proteins^76^.

In conclusion, our results highlight nucleolin as an essential host actor for the chromatin targeting of the nucleocytoplasmic export complex of vRNPs during infection. An interesting avenue to explore would now be the potential of targeting host nucleolin, as already proposed in cancer therapy^77^, and more broadly chromatin and host-associated factors, in identifying novel anti-influenza treatments.

## Methods

### Viruses and cells

IAV strain A/Moscow/10/99 (H3N2), was obtained from the French national influenza monitoring network GROG (Groupes Régionaux d’Observation de la Grippe, Lyon, France). Virus was cultivated and titered in MDCK cells and stored at −80 °C, as previously described^60^. MDCK cells were purchased from Lonza (ATCC, CCL34) and were passaged twice weekly in serum-free Ultra-MDCK medium (Lonza) supplemented with 2 mM L-glutamine (Sigma Aldrich), penicillin (225 units/ml) and streptomycin (225 μg/ml) (Lonza). Human lung epithelial cells (A549) were cultivated in Dulbecco’s Modified Eagle’s Medium (DMEM, Lonza, Biowhittaker) supplemented with 200 units/ml penicillin, 200 μg/ml streptomycin, 2 mM L-glutamine and 10% fetal calf serum (Dutscher). All cells were maintained at 37 °C with 5% CO2. For infection, viruses were inoculated at a multiplicity of infection (MOI) of 1 for 1 hpi to 24 hpi, in DMEM supplemented with 200 units/ml penicillin, 200 μg/ml streptomycin, 2 mM L-glutamine and 0.5 μg/ml TPCK-trypsin (Roche diagnostics), as previously described^17^. For viral kinetics on A549 cells, confluent cells were infected with viruses at a MOI of 2 for one hour under a minimal volume of medium at 37 °C. Cells were then overlaid with fresh medium and incubated at 37 °C. Samples of supernatants were harvested at several time points post-infection and stored at −80 °C until end point viral titration assays in MDCK cells using the Reed and Muench statistical method (TCID50/mL), as previously described^78^.

### Fluorescence immunostaining

A549 cells grown on Lab-Tek II chamber slides (ThermoScientific) were fixed with 4% paraformaldehyde in PBS for 30 min. After washing in PBS, cells were permeabilized with 0.1% Triton X-100 in PBS (PBS-T) for 15 min. Mouse monoclonal anti-nucleoprotein (17C2, VirPath laboratory)^79^, mouse monoclonal anti-nucleoprotein clone 3/1 (MAb 3/1, kind gift from R. Webster), anti-B23 (Sigma), rabbit polyclonal anti-nucleolin (#134, kindly provided by P. Bouvet) and anti-fibrillarin (Abcam, Ab5821) antibodies were used as primary antibodies in PBS-T. After incubation for 1 h, the cells were washed in PBS-T and then incubated with goat anti-mouse coupled to AlexaFluor 633 and/or goat anti-rabbit coupled to AlexaFluor 488 (Molecular Probes, Invitrogen) for 30 min, at concentrations recommended by the suppliers. Nuclei were counterstained with DNA-binding fluorochrome 4,6-diamidino-2-phenylindole (DAPI, Invitrogen). After staining, the coverslips were mounted with Fluoromount G (Cliniscience) and analyzed using a confocal laser scanning microscope (Leica). Each observation was performed on at least 20 individual cells from 5 different large fields of view. For all observations, each representative pattern was reported.

### Light microscopy observations

The Confocal Microscope Laser Scanning allows an optical section image acquisition in two dimensions X and Y. For 3D reconstruction of a stack of images, with a motorized stage, a minimum of twenty optical sections were acquired with the confocal microscope described above with a 0.1 μm z-step. JAcoP plugin for imageJ^80^ was used to highlight the colocalization of both stainings by thresholding the signal above the background. The confocal image stacks were treated by three-dimensional visualization software: Amira version 3.1 (Mercury Inc). To create three-dimensional animations and pictures, two methods were applied. Isosurface generated a geometric surface representing points of constant value as a limit between shadow and brightness of a staining. This limit was defined through an adjusted threshold. This representation was used to define the limit of the nucleus staining (DAPI). Volume rendering visualization technique was used to simulate the casting of light rays through the volume and give a 3D impression of the stained structure.

### GST pull-down assay

A/Moscow/10/99 (H3N2) NP and A/WSN/33 (H1N1) NS1, NEP open reading frames were transferred into pDEST27 plasmid (GST fusion in N-term, Invitrogen). 293T cells were transfected (JetPei, Polyplus) by these vectors (control: GST alone). Two days after transfection, cells were harvested and lysed with lysis buffer (0.5% NP-40, 20 mM Tris-HCl [pH 8.0], 180 mM NaCl, 1 mM EDTA, and complete protease inhibitor cocktail [Roche]) for 20 min on ice. Whole cell lysates were cleared by centrifugation for 20 min at 13,000 rpm at 4 °C and soluble protein complexes were purified using glutathione sepharose 4B beads (Amersham), according the manufacturer’s instructions. Beads were then washed extensively four times with lysis buffer, and pull-down proteins displayed on NuPAGE acrylamide gels before being transferred to nitrocellulose membrane. GST-tagged viral protein and endogenous nucleolin were detected using standard immunoblotting techniques with mouse anti-GST (Covance, 1:1000) and rabbit anti-nucleolin (1:1000) antibodies. All results were reproduced in at least three independent experiments.

### Immunoprecipitation

1 × 10^6^ cells were harvested 8 or 24 h after infection with the human virus A/Moscow/10/99 (H3N2). Then, cell pellets were lysed at 4 °C for 30 min in 500 μL of RIPA buffer (50 mM Tris-HCl pH 7.2, 0.1% Triton X-100, 0.1% CHAPS) that contained a cocktail of protease inhibitors (cOmplete^®^, ROCHE) and was supplemented with 150 mM NaCl. Cell extracts were centrifuged at 13000 rpm for 30 min at 4 °C. Inputs correspond to 10% of supernatants (50 μL/500 μL). Supernatants were incubated overnight at 4 °C in the presence of anti-nucleolin antibody. Immuno-complexes were immobilized for 30 min on protein A/G agarose beads (Santa-Cruz, sc-2003). Bound complexes were then washed 3 times in RIPA buffer supplemented with 150 mM NaCl 43. Then, for protein analysis, bound complexes were eluted with NuPAGE LDS sample buffer (Invitrogen, NP0008) and boiled for 5 min. Proteins were then separated on a 10% polyacrylamide/SDS gel. For viral RNA analysis, RNAs were extracted and then quantified by RT-qPCR^60^.

### Western blot analysis

The level of expression of viral or cellular proteins was assessed by western blotting. Proteins were extracted by scraping and syringing cells in 1X NuPAGE LDS buffer (Invitrogen). Ten to thirty micrograms of protein extract was separated on 10% SDS gel and then transferred to nitrocellulose membrane with a Transblot Turbo system (BioRad). The membrane was blocked by incubation 30 min in 5% non-fat dried milk in TBS-0.1% Tween buffer (TBS-T) at room temperature. The membrane was then incubated 1 hour in 1% non-fat dried TBS-T with monoclonal mouse anti-NP (CDC/IVPS, 30AUG01), goat polyclonal anti-PB1 (SantaCruz, sc-17601), rabbit polyclonal anti-NEP (kind gift from Thorsten Wolff, Robert Koch-Institute, Berlin, Germany), rabbit polyclonal anti-H3 (AbCam, ab1791), rabbit polyclonal anti-nucleolin (SantCruz, sc-13057), mouse monoclonal anti-nucleolin (SantaCruz, sc-8031). Rabbit polyclonal anti-β-tubulin (Epitomics, 1862-1) or anti-histone H3 (SantaCruz, sc8653) were used as controls. After 3 washes in TBS-T at room temperature, membranes were incubated with the appropriate secondary antibody, goat anti-mouse coupled to HRP (GE Healthcare, NXA931), goat anti-rabbit coupled to HRP (GE Healthcare, NA934) or donkey anti-goat coupled to HRP (SouthernBiotech, 6420-05), at room temperature during 30 min.

### Cell fractionation

For nucleo-cytoplasmic fractionation, 6 × 10^5^ cells were scraped into 1 mL of ice-cold PBS. After one wash in PBS, cells were fractionated with PBS, 0.1% NP-40 and protease inhibitor. One-tenth of the suspension was kept aside as whole cell lysate. After centrifugation, the supernatant was kept for the cytoplasmic fraction. The nuclei pellets were washed with PBS, and then nuclei lysed in LDS 1X (Method adapted from^81^). The protocol used for cytoplasm/nucleoplasm/chromatin150/chromatin500 fractionation, has already been described^13^,^65^. Briefly, 1.5×10^7^ A549 cells were washed and pelleted in PBS. The cell pellet was resuspended in 5 ml sucrose buffer (10 mM HEPES pH 7.9, 10 mM KCl, 2 mM Mg acetate, 3 mM CaCl2, 340 mM sucrose, 1 mM DTT and protease inhibitor), and cells were incubated 10 min on ice. NP-40 was then added to a final concentration of 0.5%, followed by vortexing for 20 sec. Lysate was triturated 6 times and vortexed for 20 sec. One-tenth of the suspension was removed for the whole extract. After centrifugation for 10 min at 4 °C at 4,500 *g*, supernatant was saved as the cytoplasmic fraction, and the nuclear pellet was washed once with 2.5 ml sucrose buffer. The nuclear pellet was then resuspended in 750 μL of nucleoplasm extraction buffer (50 mM HEPES pH 7.9, 150 mM potassium acetate, 1.5 mM MgCl2, 0.1% NP-40, 1 mM DTT and protease inhibitor), transferred to an all-glass 1 ml Dounce homogenizer, and homogenized with 60 strokes. Homogenate was then incubated 20 min at 4 °C on a rotating wheel, followed by a second homogenization with 70 strokes of Dounce, followed by a centrifugation for 10 min at 4 °C at 14,000 rpm in a microcentrifuge. The supernatant was saved as the nucleoplasmic fraction, and the pellet was resuspended in 750 μl nuclease incubation buffer (50 mM HEPES pH 7.9, 10 mM NaCl, 1.5 mM MgCl_2_, 1 mM DTT and protease inhibitor) containing 100 U/ml Benzonase nuclease (Novagen). Chromatin was digested for 10 min at 37 °C, followed by addition of NaCl to 150 mM and further incubation for 20 min on ice. Digested lysate was then centrifuged 10 min at 4 °C at 14,000 rpm, and supernatant was saved as the low-salt chromatin fraction. The pellet was resuspended in 750 μl chromatin extraction buffer (50 mM HEPES pH 7.9, 500 mM NaCl, 1.5 mM MgCl_2_, 0.1% Triton X-100, 1 mM DTT and protease inhibitor) and incubated 20 min on ice, followed by centrifugation as above. The supernatant was saved as the high-salt chromatin fraction^13^.

### Real-time quantitative RT-PCR

Viral RNA was extracted from the supernatant of infected MDCK cell cultures using the QIAmp viral RNA minikit (Qiagen), according manufacturer’s instructions. On Cellular viral RNAs were extracted using Trizol reagent method (Josset *et al*., 2008). The real-time PCR was developed to detect and quantify the number of genome copies from all subtypes of influenza type A viruses, as previously described^60^. Primers and TaqMan probe (5′FAM/3′BHQ) were designed in a highly conserved sequence of viral M gene^82^. The quantification standard was pHW2000 plasmid containing influenza A/Moscow/10/99 H3N2 M1 gene. Amplification was performed in an ABI Prism 7500 system (Applied Biosystems) using the Superscript III/Platinum *Taq* One-step qRT-PCR kit (Invitrogen). The 50 μL reaction mixture contained 5 μL extracted RNA or standard plasmid, 25 μL Superscript III/Platinum *Taq* One-step qRT-PCR reaction mix, 1 μL Superscript III/Platinum *Taq* One-step qRT-PCR enzyme mix, 0.1 μL ROX reference dye as a passive reference, 200 nmol Taqman probe, and 800 nmol of each primer. The following thermal profile was used: an initial reverse transcription at 50 °C for 15 min, followed by reverse transcriptase inactivation and DNA polymerase activation at 95 °C for 2 min and 40 cycles of amplification (15 s at 95 °C and 30 s at 60 °C). The reporter dye (FAM) signal was measured against the internal reference dye (ROX) signal to normalize the signals for non-PCR-related fluorescence fluctuations that occur from well to well. Data were collected at the annealing step of each cycle and the threshold cycle (CT) for each sample was calculated by determining the point at which the fluorescence exceeded the threshold limit.

### siRNA transfection

A pool of ON-TARGETplus SMARTpool siRNA duplexes specific for human nucleolin (si C23), siRNA J-003854-05: 5′-GCAAAGAAGGUGGUCGUU-3′, siRNA J-003854-06: 5′-GAUAGUUACUGACCGGGAA-3′, siRNA J-003854-07: 5′-CAAAUCUGCUCCUGAAUUA-3′, siRNA J-003854-08: 5′-GAAAGAAGAUGAAGUUUGA-3′ (from Dharmacon, ThermoFisher Scientific) were used to silence endogenous nucleolin expression, as previously shown^83^. Transfection was performed using Oligofectamine (Invitrogen), as previously described^60^. Two rounds of siRNA transfection were performed – with a 24 h interval - to achieve a sufficient level of nucleolin silencing. Twelve hours after the second transfection, cells were subjected to H3N2 infection at the indicated MOIs and incubated for different time periods.

### Functional analysis, network reconstruction and representation

DAVID database was used for functional annotation^84^. DAVID functional annotation chart tool was used to perform Gene Ontology biological process analysis. Gene Ontology terms with a Benjamini-Hochberg corrected p-value smaller than 5.10-2 were considered as significantly overrepresented. NP-human protein-protein interactions were retrieved from the VirHostNet database^85^. Human protein interactions were retrieved from iREF^86^. Network is visualized with Cytoscape^87^.

## Additional Information

**How to cite this article**: Terrier, O. *et al*. Nucleolin interacts with influenza A nucleoprotein and contributes to viral ribonucleoprotein complexes nuclear trafficking and efficient influenza viral replication. *Sci. Rep.* **6**, 29006; doi: 10.1038/srep29006 (2016).

## Supplementary Material

Supplementary Information
