# Supplementary material for: Nucleolin interacts with influenza A nucleoprotein and contributes to viral ribonucleoprotein complexes nuclear trafficking and efficient influenza viral replication
Source: Sci Rep. 2016 Jul 4;6:29006. doi: 10.1038/srep29006 (PMC4931502; doi:10.1038/srep29006)
Supplement: Supplementary Information [file srep29006-s1.pdf]

Supplementary data and supplementary figures

Nucleolin interacts with influenza A Nucleoprotein and contributes to viral ribonucleoprotein complexes nuclear trafficking and efficient influenza viral replication

Olivier TERRIER*, Coralie CARRON, Benoît DE CHASSEY, Julia DUBOIS, Aurélien TRAVERSIER, Thomas JULIEN, Gaëlle CARTET, Anaïs PROUST, Sabine HACOT, Denis RESSNIKOFF, Vincent LOTTEAU, Bruno LINA, Jean-Jacques DIAZ, Vincent MOULES and Manuel ROSA-CALATRAVA*

Supplementary information about viral strains

Influenza strains A/New Caledonia/20/99 (H1N1), A/Finch/England/2051/94 (H5N2) and A/Lyon/969/09 (A(H1N1)pdm2009) were obtained from the French national influenza monitoring network GROG (Groupes Régionaux d'Observation de la Grippe, Lyon, France) and the WHO collaborative center NIMR/MRC (kindly provided by Dr. Alan Hay). Virus strains A/chicken/Netherland/2003 A(H7N7) and A/Turkey/582/2006 A(H5N1) were kindly provided by Dr van den Berg (University of Louvain, Belgium) and the National Reference Center of Turkey, respectively. Influenza strains were first amplified and titered on MDCK cells (two passages) and stored at -80° C. H7N7 and H5N1 viruses were manipulated in a BioSafety Level 3 facility (VirPath, Lyon, France).

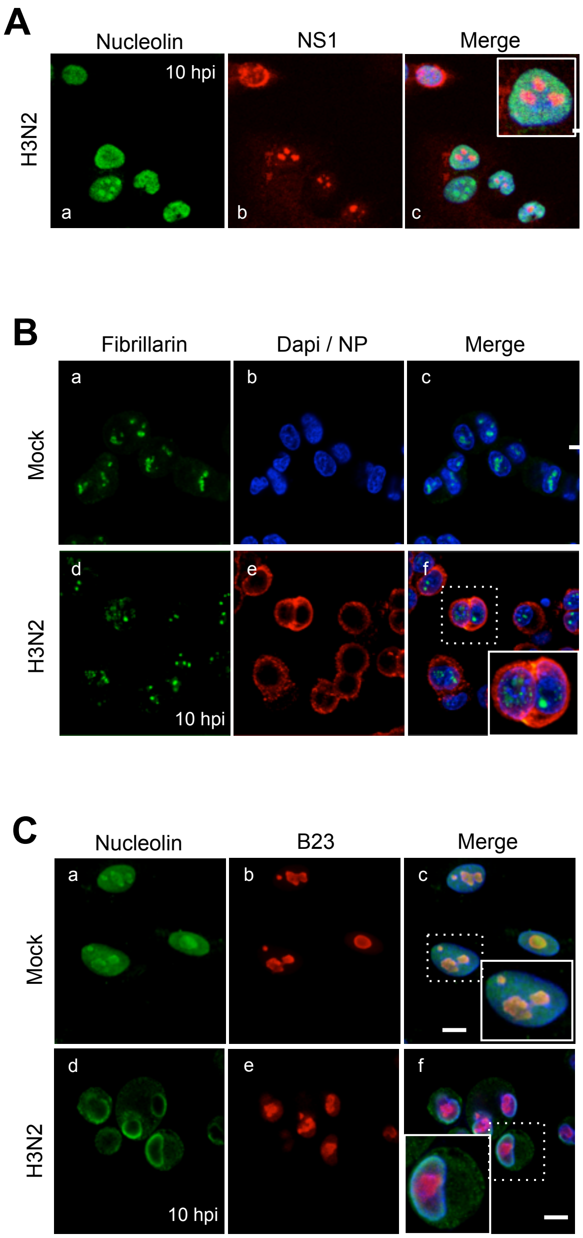

Supplementary Figure 1. Subcellular localization of NS1, fibrillarin and B23 during infection. (A) Immunofluorescent staining of nucleolin (green) and NS1 (red) in A549 cells infected with A/Moscow/ 10/99 (H3N2) virus. **(B)** Subcellular localization of fibrillarin (green) and NP (red) was characterized by immunofluorescent staining of H3N2-infected A549 cells. **(C)** Subcellular localization of Nucleolin (green) and B23 (red) was characterized by immunofluorescent staining of H3N2-infected A549 cells. Nuclei were counterstained with DAPI. Merged fluorescent signals are presented in panels c and f. Details of infected cell (arrowhead) are enlarged (inset). Scale bar = 10 μm.

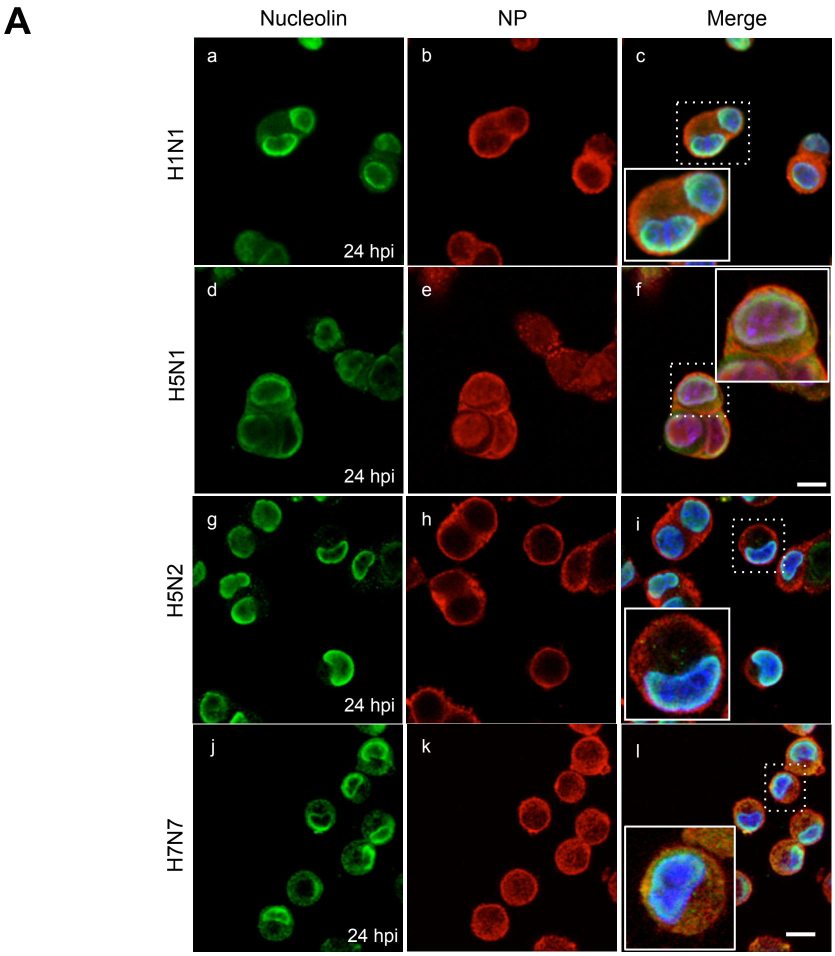

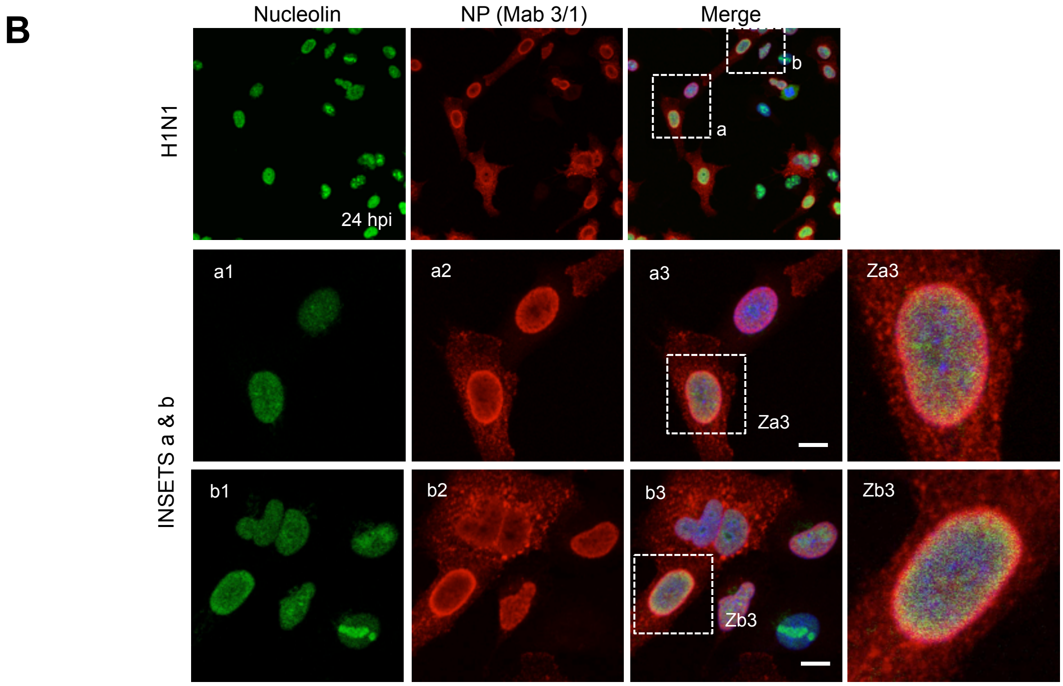

Supplementary Figure 2A & 2B

C

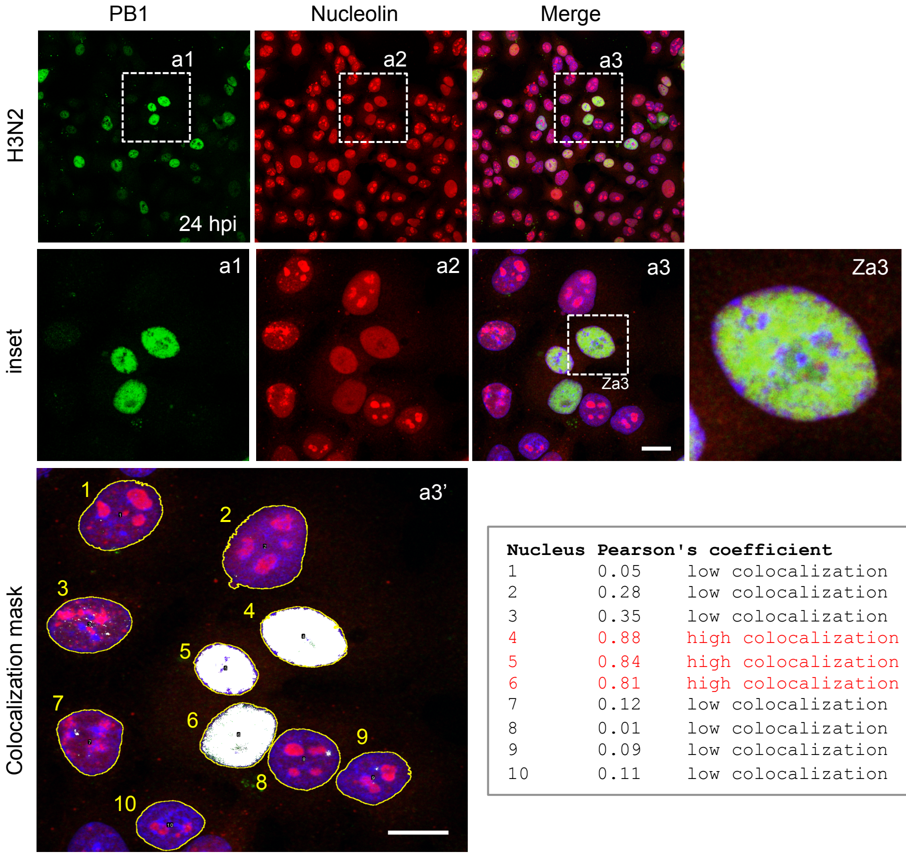

Nucleus	Pearson's coefficient	
1	0.05	low colocalization
2	0.28	low colocalization
3	0.35	low colocalization
4	0.88	high colocalization
5	0.84	high colocalization
6	0.81	high colocalization
7	0.12	low colocalization
8	0.01	low colocalization
9	0.09	low colocalization
10	0.11	low colocalization

Supplementary Figure 2C

Supplementary Figure 2. A. Human and avian influenza viruses lead to a nuclear redistribution of endogenous nucleolin. A549 cells were infected for 24 hpi with A/New Caledonia/20/99 (H1N1) (a-c), A/Turkey/582/2006 (H5N1) (d-f), A/Finch/England/2051/94 (H5N2) (g-i) or A/chicken/Belgium/2003 (H7N7) (j-l) viruses, as indicated. Cells were immunostained with anti-nucleolin (green) and anti-NP (red) antibodies. Merged confocal images are represented and details of infected cells are enlarged (c, f, i, l). Scale bar = 10 µm. **B. Influenza virus induces a nuclear polarized redistribution of nucleolin colocalized with NP/vRNPs.** A549 cells were infected for 24 hpi with A/New Caledonia/20/99 (H1N1). Cells were immunostained with anti-nucleolin (green) and a specific monoclonal antibody raised against NP (red, MAb 3/1, kind gift of Dr Webster, St Jude Children's Research Hospital) that was demonstrated to specifically recognize *bona fide* IAV vRNP complexes (Eisfeld *et al.* 2011). Similar NP-Nucleolin colocalization and subnuclear localization pattern were observed compared to Figure 1, 2 and Supplementary Figure 2A. Merged confocal images are represented and details of infected cells are enlarged (Insets a & b, zooms Za3 & Zb3). Scale bar = 10 µm. **C. Influenza virus induces a nuclear redistribution of nucleolin colocalized with viral polymerase component PB1.** A549 cells were infected for 24 hpi with A/Moscow/10/99 (H3N2) virus. Cells were immunostained with a specific antibody anti-PB1 (green) and anti-nucleolin (red). A large part of redistributed nucleolin is colocalized with PB1. Merged confocal images are represented and details of infected cells are enlarged (Inset a, zoom Za3). Scale bar = 10 µm. JAcoP plugin for imageJ was used to highlight the colocalization of nucleolin and PB1 stainings by thresholding the signal above the background. For easier visualization, colocalized pixels are colorized in white (Colocalization Mask panel, a3). ImageJ software v1.51 (Rasband, W.S., ImageJ, U. S. National Institutes of Health, Bethesda, Maryland, USA, http://imagej.nih.gov/ij/, 1997-2016) with the JACoP plugin was used to analyze the statistical correlation of two channel overlap for each nucleus. To evaluate de colocalization, Pearson's correlation coefficient was calculate (values range between 1.0 and −1.0, where 1.0 is a complete colocalization and −1.0 indicates no overlap. A coefficient above 0.7 reflects a high level of colocalization (Manders *et al.* 1993).

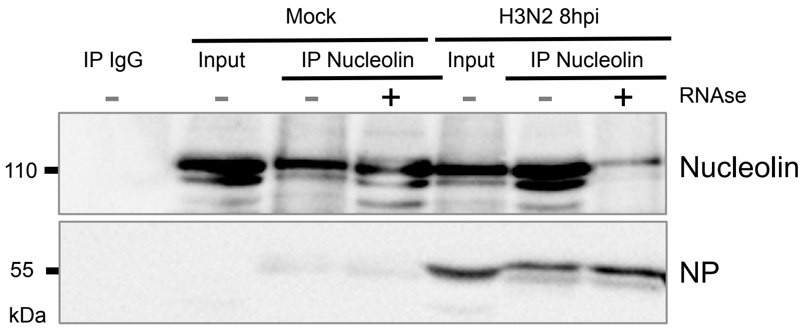

Supplementary Figure 3. **NP interacts with endogenous nucleolin: RNAse susceptibility assay.** Cells were infected during 8 hours with H3N2 (MOI 2) . Immunoprecipitations (IP) were performed on the whole cell extract with polyclonal anti-nucleolin or control IgG antibodies, as indicated. Some samples were treated by RNAse A (0.03 µg) during 15 min at room temperature. The presence of nucleolin and NP in immunopurified complexes was checked by western blot analysis. Whole cell extracts (Input) were used as control.

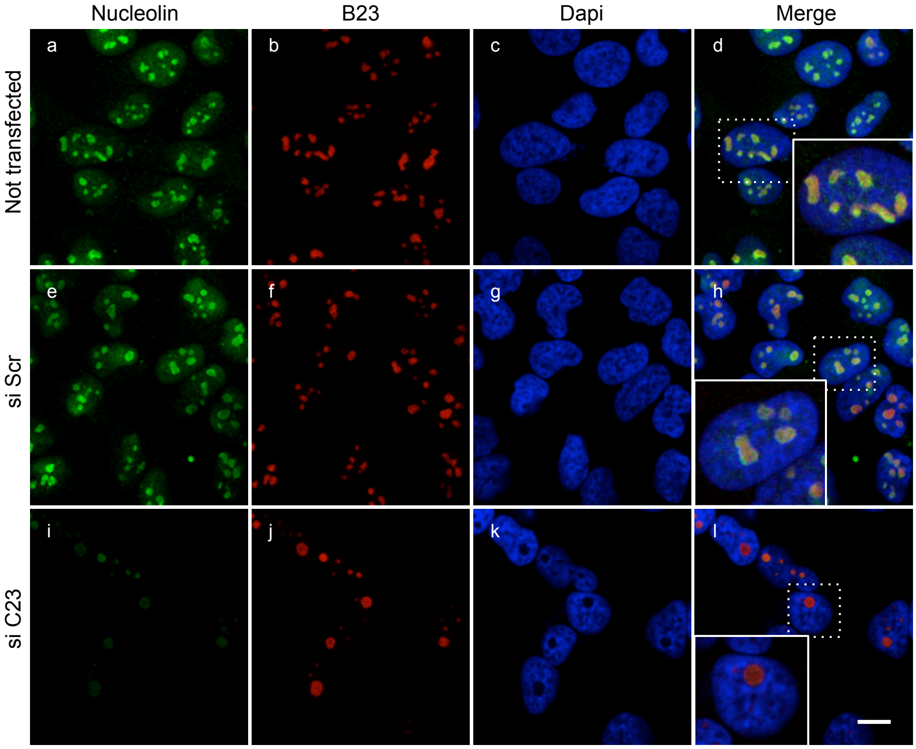

Supplementary Figure 4. Efficient nucleolin depletion by siRNAs. A549 cells were transfected or not with si-Control or si-Nucleolin. Expression of nucleolin was assessed by immunostaining (a, e, i) and nucleoli were labeled using anti-B23 antibody (b, f, j). Nuclei were counterstained with DAPI (c, g, k). Merged confocal images are represented and details of infected nucleus (arrowhead) are enlarged (d, h, l). Scale bar = 10 µm.
